# A large-scale multi-label 12-lead electrocardiogram database with standardized diagnostic statements

**DOI:** 10.1038/s41597-022-01403-5

**Published:** 2022-06-07

**Authors:** Hui Liu, Dan Chen, Da Chen, Xiyu Zhang, Huijie Li, Lipan Bian, Minglei Shu, Yinglong Wang

**Affiliations:** 1 Shandong Artificial Intelligence Institute, Qilu University of Technology (Shandong Academy of Sciences), Jinan, 250014 China; 2 Shandong Provincial Hospital Affiliated to Shandong First Medical University, Jinan, 250021 China

**Keywords:** Cardiovascular diseases, Databases

## Abstract

Deep learning approaches have exhibited a great ability on automatic interpretation of the electrocardiogram (ECG). However, large-scale public 12-lead ECG data are still limited, and the diagnostic labels are not uniform, which increases the semantic gap between clinical practice. In this study, we present a large-scale multi-label 12-lead ECG database with standardized diagnostic statements. The dataset contains 25770 ECG records from 24666 patients, which were acquired from Shandong Provincial Hospital (SPH) between 2019/08 and 2020/08. The record length is between 10 and 60 seconds. The diagnostic statements of all ECG records are in full compliance with the AHA/ACC/HRS recommendations, which aims for the standardization and interpretation of the electrocardiogram, and consist of 44 primary statements and 15 modifiers as per the standard. 46.04% records in the dataset contain ECG abnormalities, and 14.45% records have multiple diagnostic statements. The dataset also contains additional patient demographics.

## Abstract (summary)

**Table:** 

Measurement(s)	12-lead electrocardiogram
Technology Type(s)	EKG or ECG Monitor Device

## Background & Summary

Electrocardiogram (ECG) is an important tool for diagnosing heart diseases and early screening^1,2^. In recent years, as the rapid growth in leveraging wearable devices (e.g. smartwatch^3^ and smart vest^4^) for ECG monitoring, the automatic classification of ECG has become a hot topic. It is known that the ECG classification problems can be efficiently addressed by deep learning based approaches, providing that large-scale ECG data with labels of high quality are available. Successful cases include the cardiologist-level arrhythmia detection^5^ and the screening for cardiac contractile dysfunction^2^, where tens of thousands ECGs with restricted access were used in these studies.

Many ECG datasets have been published in past decades^6^, e.g., the MIT-BIH arrhythmia database^7^, the INCART database^8^, and the QT database^9^, but there are mostly dozens of ECG recordings in them, where the recordings usually have only one or two leads. In fact, such databases were mostly employed for developing models for the classification of heartbeats rather than whole ECG records. Recently, several large 12-lead ECG datasets have been made public (Table 1), e.g. the PTB-XL dataset^10,11^ and the Shaoxing People’s Hospital dataset^12,13^ respectively containing 21837 and 10646 records. Especially, the PTB-XL database, the CPSC database^14,15^, the INCART database^8^, and the Georgia database^15^, which are summarized in Table 1, have been exploited in the PhysioNet/CinC 2020 challenge^15^, leading to significant progress on real-world evaluation of the ECG classification methods. However, the existing sources of ECG are still limited for assessing the generalization ability. More importantly, ECG diagnostic statements, i.e. the label, used in the literature and competitions are not uniform. Specifically, the PTB-XL dataset adopts SCP-ECG (i.e. standard communications protocol for computer assisted electrocardiography) statements^16^ and the PhysioNet/CinC challenge uses SNOMED-CT codes^17^, and many ECG statements used by the two standards do not exactly match. In addition, more approaches just use non-standardized statements, e.g. a collection of common rhythms^5,12^. Such divergence hinders real-world application of large-scale ECG data, and may result in unfair or misleading comparison.

**Table 1:** Overview of large public 12-lead ECG datasets.

Name	# ECG	Length (seconds)	Standard	# Classes	# Patient	Single-source
CPSC database^14,15^	10330	6∼60	—	23	10330	N
INCART database^8^	74	1800	—	10	32	Y
PTB-XL dataset^10,11^	21837	10	SCP-ECG^16^	71	18885	Y
Georgia database^15^	10344	10	SNOMED-CT^17^	24	10344	N
Shaoxing People’s Hospital dataset^12,13^	10646	10	—	11	10646	Y
SPH dataset (this work)^18^	25770	10∼60	AHA^19^	44	24666	Y

In this study, we present a large 12-lead ECG dataset^18^ with standardized diagnostic statements conforming to the AHA/ACC/HRS (i.e., the American Heart Association, the American College of Cardiology, and the Heart Rhythm Society) recommendations (hereinafter referred to as “AHA standard”)^19^. Our dataset contains 25770 12-lead clinical ECG records from 24666 patients (55.36% male and 44.64% female) and, to our best knowledge, this is the largest accessible single-source ECG dataset. The data were collected from Shandong Provincial Hospital from 2019/08 to 2020/08. The sampling frequency is 500 Hz and the length of records ranges from 10 to 60 seconds. Patient demographics such as age and sex are also included. 46.04% records in the dataset contain ECG abnormalities, which are described in Chinese. The original Chinese diagnostic statements were checked by cardiologists and then converted to standardized diagnostic statements as per the AHA standard, including primary statements, modifiers and pairing rules.

The AHA standard aims for the standardization and interpretation of the ECG. It has been widely adopted in clinical scenario across the world. Compared with SNOMED-CT or SCP-ECG, which are mainly designed for medical information interchange and hence involve redundant or uncertain terms, the AHA recommendation mainly presents clinically useful statements and excludes unnecessary overlapping or vague terminology^19^. As supervised learning depends on accurate ECG labels to distinguish different ECG classes, a clearly organized and non-overlapping system of statements helps the deep learning models to efficiently learn the intrinsic ECG characteristics. Using the AHA terminology uniformly also enables valid model comparison and real-world clinical assessment.

The Shandong Provincial Hospital (SPH) database covers a wide range of ECG abnormalities and includes 44 primary statements and 15 modifiers as per the AHA standard. Especially, the separation of statements and modifiers as well as the pairing rules between them can characterize ECG more thoroughly, which is of great value to explore precise ECG analysis. 14.45% records in the database and 31.39% abnormal records have multiple diagnostic statements, providing the opportunity to develop and evaluate multi-label classification methods.

## Methods

### Data acquisition

This study was approved by the Institutional review board of Shandong Provincial Hospital. Requirement for individual patient consent was waived and the database is allowed to be shared publicly after the data were de-identified.

Original ECG records were generated at Shandong Provincial Hospital, Jinan, China during 2019/08∼2020/08. The ECG signal was recorded by the MedEx MECG-200 machine, where the A/D converter has 24-bit resolution and the unit is mV. The ECG signal has 16-bit precision and the sampling frequency is 500 Hz. Noises caused by the power line interference, baseline wander, and muscle contraction have been removed by the machine. Next, the filtered ECG signal was presented to a responsible cardiologist belonging to the department of electrocardiogram for clinical diagnosis. All cardiologists have at least three-year clinical experience before they are qualified to conduct ECG diagnosis. The ECG analysis system can automatically calculate nine ECG features for reference, which include heart rate, P wave duration, P-R interval, QRS duration, QT interval, corrected QT (QTc) interval, QRS axis, the amplitude of the R wave in lead V5 (RV5), and the amplitude of the S wave in lead V1 (SV1). The features might be inaccurate, especially when the ECG signal is abnormal. The responsible cardiologist made the final diagnosis in consideration of the patient health record.

Under the limitations that the record length should be between 10 and 60 seconds and the patient age should be larger than 18, the filtered signal and the diagnostic statements made by the doctor were then exported from the MedEx MECG-200 ECG analysis system together with the following related information from the health record database of the hospital:

- unique ID of the patient
- age and sex of the patient
- acquisition date

### Data processing

Since ECG signals have been filtered by the ECG machine and were mostly of good quality, we did not make additional processing to the signals. The ID of ECG records and patients were generated randomly, where ECG records from the same patient were still associated with the same patient ID. In order to protect the privacy of patients, the acquisition date of ECG records were shifted by a random offset for each patient^10^. When there are multiple records for the same patient, the chronological order was kept unchanged during date randomization. ECG records with missing age or sex information were excluded.

The original diagnostic statements, which were written in Chinese, mainly follow the proprietary statement set of ECG machine vendor, and also contain many inconsistent use of idioms and punctuation due to manual input. The AHA standard has 117 primary diagnostic statements under 14 categories. Each primary statement can be paired with one or more secondary statements or modifiers, which cannot be used alone. The steps to convert the original diagnostic statements to standardized AHA terminology are as follows.

1. An experienced cardiologist re-checked all original diagnostic statements and made corrections (also in Chinese). ECG records of poor quality were excluded by visual inspection at the same time.
2. We eliminated inconsistent use of idioms and punctuation as much as possible by manually converting them to uniform terminology.
3. We developed a series of translation rules mapping Chinese statements to standardized statements conforming to the AHA standard, where each original statement may correspond to multiple AHA diagnostic statements. The rules do not cover all cases since there still exists vague or clinically useless statements. The rules were revised by the cardiologist and are described in the Supplemental File 1.
4. We applied the rules to all records with original diagnostic statements. Any ECG record with untranslatable statements was excluded.

Finally, there were 25770 12-lead ECG records with standardized diagnostic statements after all steps.

## Data Records

**Fig. 1:**
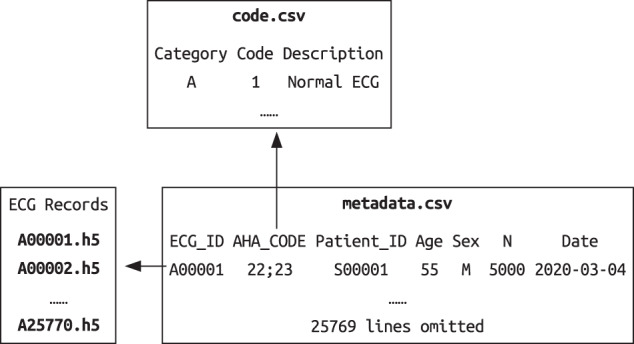
Files in the SPH database.

The SPH database includes ECG signal data, associated metadata and diagnostic statement dictionary (see Fig. 1), which are all available online at figshare^18^. Each unique ECG record was saved as a 12 × *L* array in HDF5 format with 16-bit precision, and the file was named by the associated ID (e.g. A00001.h5). The sampling frequency is 500 Hz. *L* is the number of samples and 12 is the number of leads. The order of leads is I, II, III, aVR, aVL, aVF, V1, V2, V3, V4, V5, V6. There are 25770 ECG data files in total.

The diagnostic statement dictionary file (code.csv) describes the AHA statements and codes used in the SPH dataset. As shown in Table 2, there are 44 primary statements spanning across 11 categories (see Table 3). The distribution of primary statements shown in the table is highly unbalanced and should not be viewed as the actual reflection of the population since many records were excluded for various reasons (see Methods Section). There are also 15 modifiers in Table 2, which are used to refine the meaning of core statements and cannot be used alone^19^. There are more than 40 modifiers according to the AHA standard. Some modifiers (e.g. 308 and 310) can be used with a wide range of core statements, and some modifiers can only be used with a specific category, e.g. 330–334 should be paired with statements in category M.

**Table 2:** Overview of primary statements and modifiers in the dataset.

Category	Code	Primary Statement (+ *Modifier*)	Count
A	1	Normal ECG	13905
C	21	Sinus tachycardia	725
C	22	Sinus bradycardia	2711
C	23	Sinus arrhythmia	1553
D	30	Atrial premature complex(es)	539
	*308*	+ *Occasional*	*153*
	*310*	+ *Frequent*	*125*
	*340*	+ *Couplets*	*11*
	*341*	+ *In a bigeminal pattern*	*31*
	*349*	+ *With aberrancy*	*16*
D	31	Atrial premature complexes, nonconducted	4
D	36	Junctional premature complex(es)	64
D	37	Junctional escape complex(es)	20
E	50	Atrial fibrillation	675
	*346*	+ *With a rapid ventricular response*	*210*
	*347*	+ *With a slow ventricular response*	*6*
E	51	Atrial flutter	99
E	54	Junctional tachycardia	13
F	60	Ventricular premature complex(es)	1067
	*308*	+ *Occasional*	*271*
	*310*	+ *Frequent*	*277*
	*340*	+ *Couplets*	*3*
	*341*	+ *In a bigeminal pattern*	*70*
	*342*	+ *In a bigeminal pattern*	*38*
	*350*	+ *Polymorphic*	*3*
H	80	Short PR interval	11
H	81	AV conduction ratio N:D	3
H	82	Prolonged PR interval	238
H	83	Second-degree AV block, Mobitz type I (Wenckebach)	9
H	84	Second-degree AV block, Mobitz type II	3
H	85	2:1 AV block	35
H	86	AV block, varying conduction	47
H	87	AV block, advanced (high-grade)	3
H	88	AV block, complete (third-degree)	22
I	101	Left anterior fascicular block	154
I	102	Left posterior fascicular block	6
I	104	Left bundle-branch block	84
I	105	Incomplete right bundle-branch block	1259
I	106	Right bundle-branch block	710
I	108	Ventricular preexcitation	27
J	120	Right-axis deviation	161
J	121	Left-axis deviation	138
J	125	Low voltage	322
K	140	Left atrial enlargement	19
K	142	Left ventricular hypertrophy	209
K	143	Right ventricular hypertrophy	6
L	145	ST deviation	1829
	*362*	+ *Depression*	*1024*
	*363*	+ *Elevation*	*37*
L	146	ST deviation with T-wave change	1063
L	147	T-wave abnormality	2218
	*367*	+ *Inversion*	*176*
L	148	Prolonged QT interval	24
L	152	TU fusion	9
L	153	ST-T change due to ventricular hypertrophy	88
L	155	Early repolarization	32
M	160	Anterior MI	52
	*330*	+ *Acute*	*1*
	*332*	+ *Old*	*47*
M	161	Inferior MI	120
	*330*	+ *Acute*	*2*
	*331*	+ *Recent*	*3*
	*332*	+ *Old*	*114*
M	165	Anteroseptal MI	91
	*330*	+ *Acute*	*4*
	*331*	+ *Recent*	*9*
	*332*	+ *Old*	*75*
M	166	Extensive anterior MI	7
	*332*	+ *Old*	*7*

**Table 3:** Overview of ECG categories in the dataset.

Code	Category	Count
A	Overall interpretation	13905
B	Technical conditions	0
C	Sinus node rhythms and arrhythmias	4643
D	Supraventricular arrhythmias	622
E	Supraventricular tachyarrhythmias	787
F	Ventricular arrhythmias	1067
G	Ventricular tachyarrhythmias	0
H	Atrioventricular conduction	370
I	Intraventricular and intra-atrial conduction	2195
J	Axis and voltage	612
K	Chamber hypertrophy or enlargement	229
L	ST segment, T wave, and U wave	5125
M	Myocardial infarction	260
N	Pacemaker	0

**Fig. 2:**
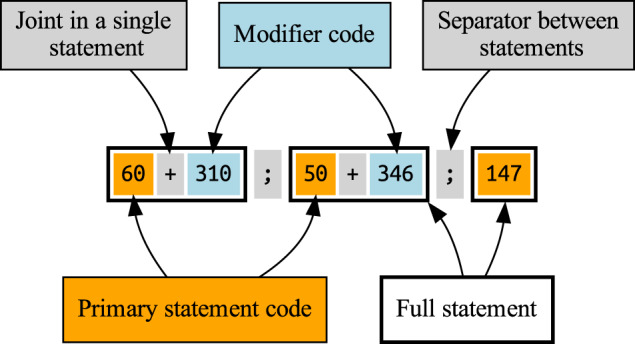
Encoded representation of AHA diagnostic statements.

In the metadata file, each line represents a unique ECG record and contains the ECG ID, the patient ID, the AHA code, the age and sex, the record length, and the acquisition date, as described in Table 4. Since an ECG record can have multiple diagnostic statements, we used the semicolon as the separator between them. Besides, each diagnostic statement consists of one primary statement and additional modifiers, and the plus sign is used to joint them. Figure 2 describes the encoded representation of multiple statements, where the order of statements and modifiers is arbitrary. The proportions of male and female in the dataset are 55.36% and 44.64%. Tables 5 and 6 show the overview of patient age and the record length respectively. Most ECG records are between 10 and 15 seconds.

**Table 4:** Metadata describing the ECG record.

Field	Type	Description
ECG_ID	String	Unique identifier for ECG
AHA_Code	String	Encoded representation (see Fig. 2) of the AHA standard
Patient_ID	String	Unique identifier for patient
Age	Integer	Age (18∼100)
Sex	String	Sex (’M’: male,’F’: female)
N	Integer	The number of sampling point
Date	String	Acquisition date

**Table 5:** Overview of patient age.

Age	[10, 20)	[20, 30)	[30, 40)	[40, 50)	[50, 60)	[60, 70)	[70, 80)	[80, 90)	[90, 100]
#Records	86	2229	5145	5110	5723	4441	2161	822	53

**Table 6:** Overview of ECG record length.

Seconds	[10, 15)	[15, 20)	[20, 25)	[25, 30)	[30, 35)	[35, 40)	[40, 45)	[45, 50)	[50, 55)	[55, 60]
#Records	24242	1141	257	71	26	15	11	3	3	1

According to Table 2, there are 13905 normal ECG records, i.e., the remaining 11865 records, 46.04% of the SPH dataset, contain ECG abnormalities. Table 7 shows the overview of the number of statements per ECG record. 14.45% records in the dataset and 31.39% abnormal records have multiple diagnostic statements. Table 8 presents the overview of the number of ECG records per patient, and 4.32% patients have more than one ECG record.

**Table 7:** Overview of number of statements per ECG record.

#Statements	1	2	3	4	5	6
#Records	22046	2936	665	109	12	2

**Table 8:** Overview of number of ECG records per patient.

#Records	1	2	3	4	5
#Patients	23600	1033	29	3	1

## Technical Validation

**Fig. 3:**
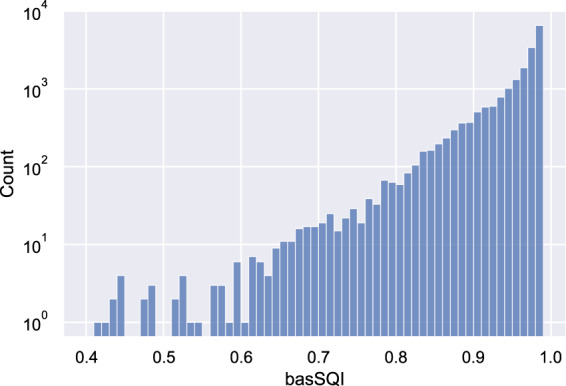
Distribution of basSQI of all ECG records.

**Fig. 4:**
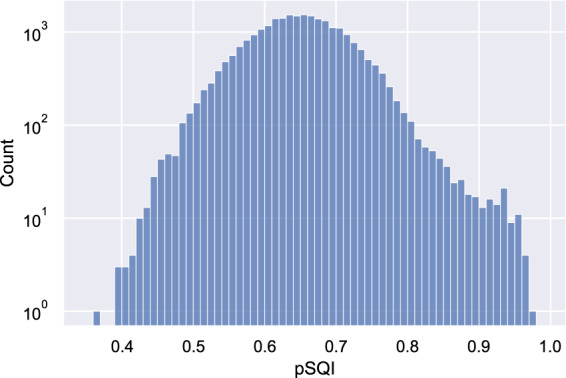
Distribution of pSQI of all ECG records.

**Fig. 5:**
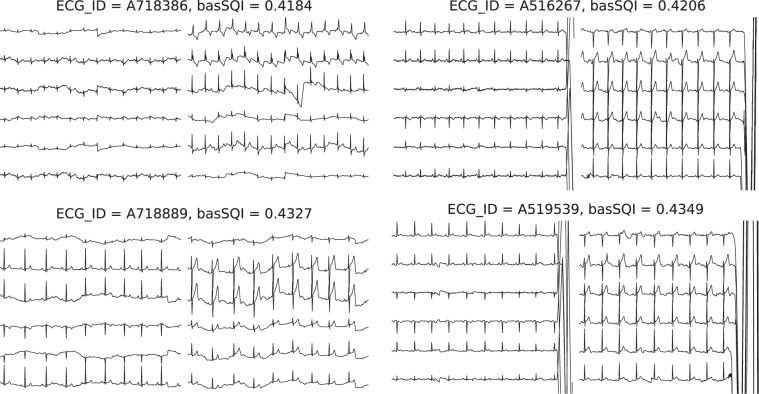
ECG signals with lowest basSQI values. The length is 10 seconds.

To validate the quality of ECG records, after all the steps described in Methods Section, we conducted signal quality assessment for original ECG records using two signal quality indices^20^, basSQI and pSQI representing the relative power in the baseline and the QRS complex respectively. For each record, the index was first calculated for 12 leads separately and then averaged. Next, we checked ECG records with low SQI values to make sure their quality is still acceptable. Figures 3 and 4 visualize the distributions of basSQI and pSQI of ECG records in the database, which have a minimum of 0.418 and 0.370 respectively. Specifically, ECG records whose SQI values are close to the minimum were reviewed and no significant quality defect was found. Figure 5 shows four records with lowest basSQI values. It is worth pointing out that ECG records containing one or two noisy leads or short corrupted segments, as shown in Fig. 5, were not rejected as long as reliable diagnosis can be made.

**Fig. 6:**
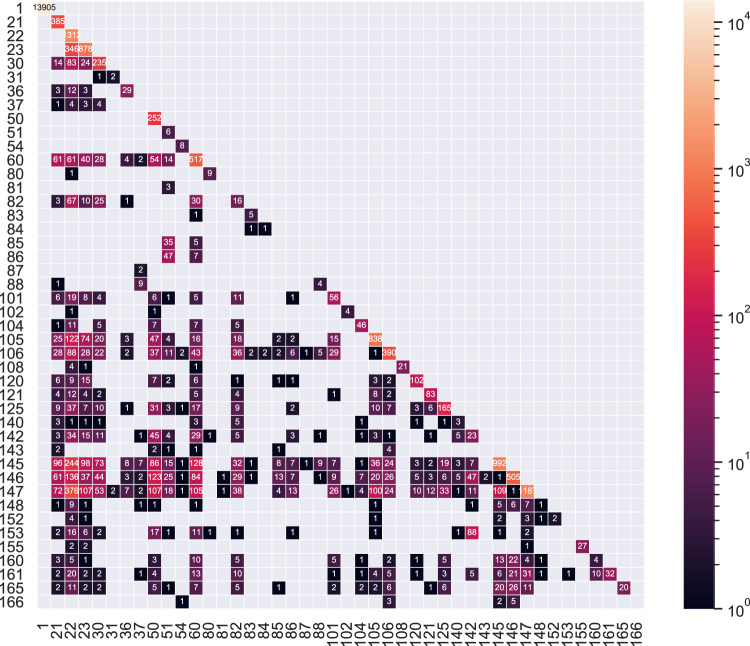
Co-occurrence matrix of primary statements. The diagonal element represents the number of records labeled only by the specified statement.

Since an ECG record may have multiple diagnostic statements, we computed the co-occurrence matrix for primary statements to show the co-occurrence relationship (see Fig. 6). For a specific primary statement, the diagonal element represents the number of records labeled only by the specified statement, and other element in the same row means the number of records labeled by both statements. If two statements are not likely to happen at the same time in clinical practice, e.g. atrial fibrillation and other sinus rhythms, the matrix can indicate whether such records exist in the database intuitively. Since co-existing statements may describe different ECG intervals, the cardiologist revised the suspicious records to make sure the statements are correct. In addition, normal ECG records should not have statements indicating abnormalities, which is verified by the first column, thus the matrix was also used for quality control purposes.

AHA standard includes various pairing rules. As this dataset contains 44 primary statements as well as 15 modifiers, we make sure that each modifier is pairing correctly with the primary statement by checking all 25 combinations (see Table 2).

## Usage Notes

The ECG data are stored in HDF5 format, a platform-independent format designed for data storage and widely supported by scientific software and programming languages. Python code for loading the ECG data and processing diagnostic codes as well as the metadata is provided at figshare^18^.

**Fig. 7:**
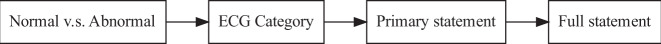
ECG classification tasks from coarse level to fine level.

The hierarchy of ECG terminology presented by the AHA standard is well-organized. 117 primary statements belong to core statements, and most ECG classification in the literature focus on this level only. Considering all kinds of AHA statements and various real-world use cases, we suggest four tasks from coarse to fine levels for the usage of SPH dataset (see Fig. 7).

The first task is the classification of normal ECG and abnormal ECG, which account for 53.96% and 46.04% in the dataset respectively. The detection of ECG abnormalities is useful in daily ECG monitoring. The remaining three tasks are at the levels of ECG category, primary statement, and full statement respectively, corresponding to the AHA standard, and all of them are multi-label classification. In addition, to avoid incorrect dataset partition (e.g. records from the same patient included in both training and testing sets) and improve comparability of models trained on the dataset, we provide the Python code at figshare^18^ for dataset splitting.

## Supplementary information

Supplemental File 1

## Data Availability

The Python code for reading the ECG data, attributes and diagnostic code dictionary, evaluating the signal quality, and dataset partition is available in figshare^18^.
