# Supplementary material for: A large-scale multi-label 12-lead electrocardiogram database with standardized diagnostic statements
Source: Sci Data. 2022 Jun 7;9:272. doi: 10.1038/s41597-022-01403-5 (PMC9174207; doi:10.1038/s41597-022-01403-5)
Supplement: Supplementary file 1 — Supplemental File 1 [file 41597_2022_1403_MOESM1_ESM.pdf]

Table 1: Translation rules mapping Chinese ECG diagnostic statements to AHA statement codes.

Diagnostic statement	AHA_Code
正常心电图	1
窦性心律	20
窦性心动过速	21
窦性心动过缓	22
窦性心律不齐	23
窦性心动过缓伴不齐	22;23
房性早搏	30
偶发房性早搏	30+308
频发房性早搏	30+310
房早未下传	31
房早伴差传	30+349
房早连发	30+340
房早二联律	30+341
房早三联律	30+342
多源房早	30+350
交界性早搏	36
交界性逸搏	37
心房颤动	50
房颤伴快速心室率	50+346
房颤伴慢速心室率	50+347
心房扑动	51
房扑呈 1:1 房室传导	51;81
房扑呈 2:1 房室传导	51;85
房扑呈 3:1 房室传导	51;81
房扑呈 4:1 房室传导	51;81
房扑伴不规则房室传导	51;86
交界性心动过速	54
室性早搏	60
偶发室性早搏	60+308
频发室性早搏	60+310
室早连发	60+340
室早二联律	60+341
室早三联律	60+342
多源室早	60+350
多型室早	60+344
P-R 间期缩短	80

P-R 间期延长	82
I 度房室传导阻滞	82
II 度 I 型房室传导阻滞	83
II 度 II 型房室传导阻滞	84
高度房室传导阻滞	87
III 度房室传导阻滞	88
左前分支阻滞	101
左后分支传导阻滞	102
完全性左束支传导阻滞	104
完全性左束支传导阻滞合并电轴左偏	104;121
完全性右束支传导阻滞	106
完全性右束支传导阻滞合并左前分支阻滞	106;101
完全性右束支传导阻滞合并左后分支阻滞	106;102
不完全性右束支传导阻滞	105
不完全性右束支传导阻滞合并左前分支阻滞	105;101
不完全性右束支传导阻滞合并左后分支阻滞	105;102
预激综合征	108
心室预激波	108
QRS 电轴右偏	120
QRS 电轴左偏	121
低电压	125
左心房肥大	140
右心房肥大	141
左室肥大	142
左室肥大伴 ST-T 改变	142;153
右室肥大	143
ST 段改变	145
ST-T 改变	146
ST 段抬高	145+363
ST 段抬高，急性前壁心肌缺血	145+363+220
ST 段下移	145+362
T 波改变	147
T 波倒置	147+367
T 波倒置，前壁心肌缺血	147+226+367
非特异性 ST-T 改变	146+321
Q-T 延长	148
T-U 融合	152
早期复极综合征	155
前壁心肌梗死	160

亚急性前壁心肌梗死	160+331
陈旧性前壁心肌梗死	160+332
急性前壁心肌梗死	160+330
广泛前壁心肌梗死	166
急性广泛前壁心肌梗死	166+330
亚急性广泛前壁心肌梗死	166+331
陈旧性广泛前壁心肌梗死	166+332
前间壁心肌梗死	165
急性前间壁心肌梗死	165+330
亚急性前间壁心肌梗死	165+331
陈旧性前间壁心肌梗死	165+332
下壁心肌梗死	161
急性下壁心肌梗死	161+330
亚急性下壁心肌梗死	161+331
陈旧性下壁心肌梗死	161+332
